# Results of Mentoring in the Psychosocial Well-Being of Young Immigrants and Refugees in Spain

**DOI:** 10.3390/healthcare9010013

**Published:** 2020-12-24

**Authors:** Anna Sánchez-Aragón, Angel Belzunegui-Eraso, Òscar Prieto-Flores

**Affiliations:** 1 Social & Business Research Laboratory (SBRlab), Rovira i Virgili University, 43002 Tarragona, Spain; angel.belzunegui@urv.cat; 2 Medical Anthropology Research Center, Rovira i Virgili University, 43002 Tarragona, Spain; 3 Department of Pedagogy, School of Education & Psychology, University of Girona, 17071 Girona, Spain; oscar.prieto@udg.edu

**Keywords:** youth mentoring, immigrants, social inclusion, psychosocial well-being, youth health, acculturative stress

## Abstract

This study examined the change processes associated with the Nightingale project, a community-based mentoring programme whose aim is to promote the social inclusion of minors of immigrant origin. A pre-test–post-test study was conducted on a group of 158 young immigrants between the ages of 8 and 15, in which the influence of the mentoring programme on the youths’ psychosocial well-being was measured. Non-parametric tests were used to calculate the results before and after mentoring, comparing the results over a six-month period and controlling for sex and age. The analyses reflected associations between mentoring and improvements in specific aspects of the emotional well-being of young immigrants and highlighted the potential of mentorships to cushion the stressful events they are subjected to in the process of adapting to a new social reality.

## 1. Introduction

The present study evaluates the effectiveness of the Nightingale mentoring programme in Spain, whose main objective is to promote the social, cultural and linguistic inclusion of students of immigrant origin. The project, which is part of the European-wide Nightingale Mentoring Network, was first carried out in 1997 at the University of Malmö, Sweden; since its success, it has been implemented by twenty-seven other universities in six European countries and one in Africa [1]. The programme is based on the voluntary action of university students who, during the school year, act as mentors of immigrant, refugee or irregular status children and adolescents with the aim of helping them navigate the new educational context and develop a sense of belonging that enables them to find their place within the new country. One key characteristic of the Nightingale project is that both sides benefit: the older person also learns from the younger one and improves their intercultural competence [2].

The Nightingale project is a community-based mentoring programme that fosters relationships of trust between university students and minors of foreign origin with the goal of promoting the positive development of migrants at risk of social exclusion. Throughout the school year, the volunteer mentors work following a one-on-one mentoring scheme to support the youths in their process of integration in the new community. The meetings, which are held weekly, take place outside school hours and last approximately three hours in which the mentor accompanies the minor in acquiring necessary basic elements, such as language and educational guidance. They also aim to expand mentees’ social support network outside the school by organising activities with other mentees who are in the same situation and help them to discover the cultural and leisure activities that the new environment offers. For example, some branches of the Nightingale programme teach their mentors in the training sessions how to establish meaningful conversations with their mentees in order to identify adults from their everyday life (i.e., teachers) who can become natural mentors. They are also encouraged to speak in Catalan or Basque if this is not an obstacle for communication or for establishing a close relationship. Through this relationship, the programme helps to support greater social participation of the minors as it helps them to make use of social and community resources, such as public transport and the local library. This is especially beneficial for minors with introverted personalities, so that their mentoring relation provides them with opportunities to reduce their isolation and be more sociable.

Primary and secondary school teachers are in charge of selecting the minors who will receive mentoring. The selection criteria are that (a) they have arrived recently or are of foreign origin but are capable of minimally communicating with others; (b) they lack relationships with adults in their new environment; (c) they are the first generation in their family to have access to university; and (d) they do not start from an extreme level of vulnerability that requires the intervention of a professional rather than a volunteer [1]. With regard to the selection of the mentors, given that the protection of young people is critical, this was carried out through an interview or questionnaire in some cases; and, if selected, an intensive training course of about ten hours was provided [1,3]. The training sessions include the treatment of the cultural diversity and social integration of adolescent immigrants, as well as the collection and exchange of previous mentoring experiences so that the participants are aware of the challenges involved. The primary goal is to build a supportive friendly relationship that facilitates meaningful conversation. For this to happen, the volunteer students are encouraged to mention in their meetings important issues related to the future, the process of integration in the new community or the mentees’ relationship with their parents [1]. In return for being mentors, the volunteers receive between one and four ECTS (European Credit Transfer and Accumulation System) credits, depending on the university.

The Nightingale project has been recognised as a successful initiative for vulnerable migrants in Europe [4] and in fact, in Spain, there are already several regions in which it is being carried out, such as Barcelona, Tarragona, Girona, Guipúzcoa and Navarra. The results of this study complement previous evaluations that attribute positive impacts to the programme in terms of academic attitudes—behaviour and dedication to study—and educational expectations, as well as improvements in the minors’ communication skills and self-esteem [1,3].

This article gathers together, first of all, the main contributions of the scientific literature regarding the most significant benefits of social support during the migration process. Specifically, it addresses those aspects of mentoring that influence the subjective well-being and health of young people of immigrant origin. Second, it presents the research methodology, followed by a description of the extracted results. The discussion section then offers an interpretation of the findings in light of the available evidence. Finally, the limitations and conclusions of the study are presented.

## 2. Mentoring with Vulnerable Adolescents

### 2.1. The Function of Integration in a New Social Reality

There are many scientific studies that highlight the role that non-parental adults can play in the social inclusion of the most vulnerable collectives. The need to improve the support network of people at risk of exclusion has given rise in recent years to formal mentoring programmes, which provide a safe context for the development of relationships. The fundamental goal is to provide support for people who do not usually come into contact with natural mentors or that lack the necessary social skills to identify and form this type of supportive relationship on their own. With this goal in mind, for the formal mentoring the programme recruits adult volunteers to provide friendship, guidance and support to those who most need it. While it is true that the mentoring that arises from natural networks of support usually lasts longer, with stronger affective bonds that last an average of almost a decade [5], formal mentoring has proven to be especially beneficial for those specific groups of minors and adolescents facing risks and adversity. However, not all people have a support network that can help them succeed. Erikson, McDonald and Elder [6] draw attention to the scarcity or absence of social capital in at-risk groups, some from poor communities where low levels of support can be exacerbated by an inability of adults to effectively connect the young people to opportunities that may significantly improve their life journeys.

The results of the scientific evaluations that have been carried out to date highlight that youth mentoring is related to better physical and mental health, as well as to fewer symptoms of depression or suicidal thoughts among young people [5]. An extensive body of research shows that the programmes increase the self-esteem [7,8] and personal satisfaction of the mentee [3] in addition to reducing their levels of stress and anxiety, which is particularly important when it comes to immigrants or refugees, some of them in an irregular situation, who have just arrived to the country and are under great stress due to the integration process in which they find themselves [9,10]. The scientific literature has also reported good results regarding social competencies and communication skills [8]. Studies show that a close bond with the mentor causes participants to develop more positive expectations about relationships with people in their environment, with their parents or friends, and to improve their quality [8,11,12]. This emotional stability is associated with improvements in academic performance and self-efficiency [13], as well as a reduction in behavioural problems such as aggression [14], substance use [15] or delinquent behaviour [16]. As a result, mentoring programmes for minors and young people have grown in popularity as an effective intervention strategy for the social inclusion, health and well-being of the most vulnerable collectives [17]. In the United States, an estimated 2.5 million children and adolescents maintain formal mentoring relationships each year [18].

### 2.2. Social Support as a Source of Protection Against Stress

The migration process entails an adaption effort whose intensity can be an additional risk factor for developing mental, social or behavioural health problems. The acculturative stress related to cultural differences and perceived racial discrimination in the new country increases the probabilities of young immigrants suffering from anxiety, depression and other mental conditions [19,20], such as eating disorders [21] and alcohol consumption [22]. Refugees, in particular, run a greater risk of suffering symptoms of posttraumatic stress due to exposure to war, long-term persecution or the loss of relatives both in their country of origin and during displacement [23]. Accumulated research has shown that young immigrants and refugees usually have worse mental health than the host population as a result of the challenges of the adaptation process which, as well as forcing them to adhere to new customs and sociocultural codes, is accompanied by changes and distress that occur on an individual level, such as separation from the family as well as from friends and close relationships, the loss of their ethnocultural surroundings, the learning of a new language and the prolongation of uncertainties about their migration status.

These young people suffer from specific vulnerabilities and stress that require people to support them in a lasting way and remain at their side during the adaptation process. Mentoring initiatives are designed to facilitate practical help and advice that enables the minors to cope as best as they can in the new environment; for example, introducing them to local cultural characteristics and recreational opportunities. In Australia, Singh and Tregale [24] observed how mentoring improved participants’ access to the networks of social and cultural capital networks. During the programme, the mentors acted as agents for the empowerment of immigrant minors in a process through which the mentees acquired tools to gain autonomy in the new country and achieve life goals. In Europe, Raithelhuber [25] reached similar results and found that mentoring served as a springboard for the efficient integration of the youths participating in the study, unaccompanied refugees who, thanks to their mentors, saw their opportunities for civic participation increase. In the North American context, other studies corroborated both the more practical and instrumental effects of mentoring, such as learning the new language, improving academic performance or the acquisition of social skills [26], and the more emotional effects. For example, Gonzales, Suárez-Orozco and Dedios-Sanguineti [10] recognised that mentoring is especially helpful in reducing the levels of stress and anxiety of illegal immigrant adolescents, which also has a direct impact on reducing school dropout. However, there are still few empirical studies that shed light on the impact of mentoring programmes on the social inclusion of immigrant and refugee adolescents. This study aimed to address this knowledge gap in this particular area.

### 2.3. Relationship with Parents and Family Dynamics

Empirical evidence shows that minors adapt to the new cultural context faster than their parents. The integration process for adult immigrants or refugees is fraught with difficulties given the deterioration in cognitive flexibility and their solidified ethnicity [27]. This acculturation gap between parents and children increases generational conflicts, sometimes due to the concern of the elderly for the loss of the culture of origin. Perreira, Chapman and Stein [28], through their interviews with Latino migrants with residency in the United States, highlighted parents’ concern and unease over the dissolution of cultural heritage and their sons and daughters’ adoption of the values, norms and conduct of the majority culture. These differences in the adaptation process leave immigrant minors at a crossroads: on the one hand, they must learn the language and the cultural codes of the new country if they wish to thrive and be successful at school; while on the other hand, they are forced to retain the culture of origin in order to ward off conflict and continue maintaining positive relationships with their co-ethnic families and friends [29,30].

Paradoxically, the rapid acquisition of the vehicular languages causes these young people to take on responsibilities similar to those of the adults in the household, like answering phone calls, helping parents to fill out a job application or acting as interpreters in the doctor’s surgery. In primary education, there is a strong assimilative pressure in which the language of instruction is Spanish, and only in the case of the autonomous communities, Catalan and Basque. As a result, the acquisition of vehicular languages is usually even faster in minors. One of the terms that has been used to describe this phenomenon is that of child language brokering [31], which is associated with higher levels of child stress and an increase in family conflicts [32]. In this context, or in others where the young people do not receive sufficient support from their parents due to the family structure or circumstances, mentoring can be a valuable source of support. The research of Dolan et al. [33] on the Big Brothers Big Sisters programme in Ireland supports this theory. After a study of around one hundred and fifty minors, the researchers discovered that mentoring was more beneficial for young people of single-parent families than for those who lived with both parents. After eighteen months, the difference in outcomes between the two groups with regards to perceived support decreased steadily, demonstrating that mentoring can make up for the support that some young people lack.

### 2.4. Adolescents at Risk, Mentoring and Educational Success

Some research has shown the benefits of mentoring with regards to learning and continuity of studies. For example, in the North American context, Erikson, McDonald and Elder [6] observed how young people from disadvantaged backgrounds who received the support of a non-parental adult were more likely to enrol in a university. The availability of sources of help has been associated with academic success [34,35]. In particular, the presence of a mentor has shown that it can reduce a student’s absenteeism, promote a sense of belonging to the school and improve educational expectations [36]. Herrera, Grossman, Kauh and McMaken [13] emphasise that learning difficulties and the intensity with which they experience situations of risk and precariousness can push immigrant minors to processes of demotivation and disaffection with school that lead to educational failure. However, the accumulated evidence indicates that mentoring relationships can reverse this situation and constitute a strong antidote to the dropout rate of immigrant minors and adolescents.

Young people are able to glimpse their future selves in their mentors who, with their experience, provide them with clear and generally positive messages about their own possibilities of success [37]. In the aforementioned research, Singh and Tregale [24] observed how the school motivation of young immigrants and refugee families improved after mentoring, as they saw in their older mentors where higher education could lead them to in the future. In a similar vein, based on their extensive literature review, Larose and Tarabulsy [38] indicated that youth mentoring—even though it takes place outside the educational context—helps to improve students’ academic attitudes and their relationship with school, which can have a significant positive impact on student performance. In fact, the results of the first representative survey in the United States with adolescents, collected in The Mentoring Effect [39], show that young people at risk of exclusion who have a mentor are 20% more likely to complete their studies that those who do not. The study of Herrera et al. [13] on the Big Brothers Big Sisters programme shows that mentoring improves the school performance of vulnerable minors, most of them from ethnic minorities, and their perception of their own academic skills.

### 2.5. Perceived Racial Discrimination and Cultural Mistrust

There are many studies that show the negative psychological and social effects of racial discrimination in the immigrant population [40], especially in those that come from racial and ethnic minorities [41]. The empirical evidence indicates that stress stemming from the feeling of discrimination can—in addition to causing cardiovascular disorders [42]—cause symptoms of anxiety, depression, psychological distress or suicidal ideation that directly affect mental health [43]. Young people who have suffered racialisation first-hand also tend to have greater cultural mistrust of other adults, which can negatively affect different areas of their personal lives. For example, Cooper and Sánchez [44] observed how discrimination, as a subjective perception, with respect to the host society, aggravated the cultural mistrust of Latino boys, which led them to devalue the educational process and lower their academic performance. It has been shown that mentoring can help young people to establish positive intercultural relationships [3,45] and reduce the level of stress stemming from racial discrimination [46]. For this to be possible, the need for culturally aware mentors has been highlighted [47]. It has been demonstrated that adult volunteers who have greater intercultural competencies are more likely to develop greater emotional proximity with the minor and establish a positive relationship that enables the young person to benefit from it [48].

## 3. Materials and Methods

### 3.1. Purpose and Design

The purpose of this study was to evaluate the changes in specific aspects of emotional well-being derived from the Nightingale mentoring programme aimed at adolescents of foreign origin in the primary and secondary schools of Barcelona, Tarragona, Girona, Guipúzcoa and Navarra. In order to contribute to the literature on social mentoring, this study examined the effects of mentoring on the psychological well-being of minors of immigrant origin.

The evaluation of some aspects of participants’ emotional well-being resulting from applying the programme was performed through a pre-test–post-test design of a single non-randomised group [49,50], with a six-month difference between the first and second data collections on the study’s different areas of focus. A longitudinal strategy was chosen through a dynamic comparison, including the repetition of response measures.

Specifically, this assesses aspects related to the mental health of the mentee, their self-esteem and personal satisfaction, as well as their perceptions of social support and racial discrimination during their integration process in the host country.

### 3.2. Sample

It was not possible to randomise the selection of participants due to the small starting population size, and also because participation required voluntary consent.

The study had a sample of 158 students, between 8 and 15 years of age, 79 (50%) of whom were girls, and with an average age of 12.17 years (Std. Deviation = 1.79). The participants came from different countries, although the majority were from Morocco, Honduras, Gambia, India and the Dominican Republic. Sixty-two percent were first generation immigrants and 38% second generation. Among them, 17.7% arrived in Spain the same year that they began their participation in the programme (2018), 19.6% arrived in 2017, 11.4% between 2014 and 2016, and 13.3% over five years ago (between 2007 and 2013). Moreover, 48.7% of the participants were under 12 years of age; and 51.3% were between 12 and 15. At the time of this study, 51.3% of the minors lived in a nuclear household with their parents; 12% in a household with more than two nuclear families (with extended family); 19.6% in a single-parent family; 3.8% with grandparents or other relatives; and 12.7% in a single-parent household but with other relatives. Participants’ characteristics are depicted in Table 1.

The programme began at the start of the 2018–2019 school year, and the outcomes of the participants were observed during that year. Before the evaluation, the informed consents were given for each father/mother and/or legal guardian, and only after obtaining the corresponding permission were the tests administered.

### 3.3. Study Variables

The Youth Environment Stressors Index [51] collects the joint scores of the following items, which all refer to events that happened in the previous year: someone close dying; moved or changed home; changed school; a close friend moved away; picked on or bullied at school or in the neighbourhood; parents separated or divorced; parent/guardian stopped working or lost a job; and brother/sister dropped out of school. All the items were categorised as: 1 = Strongly disagree; 2 = Disagree; 3 = Agree; 4 = Strongly agree.

The following questions were used as indicators of stressors: (a) Suddenly scared for no reason?—with answers: 0 = No, 1 = Yes, sometimes; 2 = Often; (b) Feeling blocked in getting things done?—with answers: 0 = No, 1 = Yes, sometimes; 2 = Often.

It is not properly a scale but rather a variable resulting from the summation of a number of items that give each individual a total count of environmental stressors. The resulting variable (total number of environmental stressors endorsed) is used as an ordinal variable and does not need to present the required properties on a scale that tries to measure a coherent dimension [51].

The Cultural Mistrust Index [52] was generated from the summation of the scores to different items: You should be suspicious of the native population from the beginning; You can trust the native population like members of your own group; The native population tend to keep their promises less; The native population tend to be honest with us; The native population tend to say one thing and do another; You should be cautious about what you say because it will be used against you; The native population usually tend to keep their word; The native population tend to feel superior to us. All the items were categorised as: 1 = Strongly disagree; 2 = Disagree; 3 = Agree; 4 = Strongly agree.

This index was developed from a proposal contained in Benkert, Peters, Clark and Keves-Foster [52], who used a more extensive instrument with high internal consistency (Cronbach’s alpha = 0.89). In our sample, Cronbach’s alpha was 0.89.

The Social Support Index [53] is the result of the summation of the scores of the following items: I have the social support of teachers; I have the social support of friends; I have the social support of parents/guardians; I have social support outside the family and school. All the items were categorised in the same way: 1 = Strongly disagree; 2 = Disagree; 3 = Moderately disagree; 4 = Neutral; 5 = Moderately agree; 6 = Agree; 7 = Strongly agree.

This index was developed from various scales used by Wang and Eccles [53], such as the Teacher Social Support (with a Cronbach’s alpha of 0.80), the Peer Social Support (with a Cronbach’s alpha of 0.82) and the Parent Social Support (with a Cronbach’s alpha of 0.77) scales. In our study, Cronbach’s alpha obtained a value of 0.53.

The Self-Esteem Index [54] consists of 10 items that allude to global feelings of self-esteem and self-perception. Five of the items are positive statements: I feel that I’m a person of worth, at least on an equal level with others; I feel that I have a number of good qualities; I am able to do things as well as most other people; I take a positive attitude toward myself; On the whole, I am satisfied with myself—and five others that are negative statements: All in all, I am inclined to feel that I am a failure; I feel I do not have much to be proud of; I wish I could have more respect for myself; I certainly feel useless at times; at times I think I am no good at all. All the items were categorised as: 1 = Strongly disagree; 2 = Disagree; 3 = Agree; 4 = Strongly agree.

The psychometric tests of the scale presented by Rosenberg [54] reflected a reproducibility coefficient of 0.92, which indicates excellent internal consistency. Test–retest reliability over a two-week period revealed correlations of 0.85 and 0.88, which indicates stability. In our analysis, Cronbach’s alpha was 0.73.

The Academic Self-Efficacy Index [55] consists of the following items: I feel like I do well in school; It is easy for me to learn most things; Even when I study hard, I cannot do well on tests; When I try hard, I can learn most things; I can get good grades even when I do not try hard; There is no way a student like me can get good grades; and I think I am a smart person. All the items were categorised as: 1 = Very false; 2= More false than true; 3 = More true than false; 4 = Very true.

In the consistency analysis performed by Owen and Froman [55], for reliability estimation, alpha internal consistency estimates were 0.90. In the analysis of our data, Cronbach’s alpha was 0.54.

The Cognitive Engagement Index [56] consists of the following items: With regards to learning at school, how often do the following occur: I enjoy learning new things; I get bored easily with school work; I feel good when I learn something new even when it is hard and I do homework whenever it is required. All the items were categorised as: 1 = Never; 2 = Few times; 3 = Most of the times; 4 = Always. This index was a composite of the variables validated by previous studies, such as Finn and Voelkl [56], observing compliance or the non-compliance of the aforementioned school requirements. This index had a Cronbach’s alpha of 0.43.

### 3.4. Analysis Performed

The lack of randomisation has led to non-parametric analyses to measure the effects. *Wilcoxon (Z) t*-tests—a non-parametric test equivalent to the Student’s *t*-test—were carried out to determine whether the programme had an effect on the adolescents. This test was used because the distributions failed to meet the criteria of normality and homoscedasticity, in addition to being based on small subsamples. Most of the measurements that were taken correspond to ordinal variables with few ranges. For each of the *Z* tests, Cohen’s *d* was calculated as the effect size coefficient [57] (p. 12) [58].

## 4. Results

The results show that the mentored students that took part in the project reduced the impact of stressful life experiences measured through the Youth Environment Stressors Index (Table 2). During the course of the programme (pre-post), they significantly reduced their levels of environmental stress (*Z* = 5.931; *p* = 0.000; *d_Cohen_* = 1.07). This difference is even greater in the girls (*Z* = 4.679; *p* = 0.000; *d_Cohen_* = 1.238) than in the boys (*Z* = 3.666; *p* = 0.000; *d_Cohen_* = 0.906). The differences are also significant if controlled by age.

In addition to environmental stressors, another source of anxiety in children is the interaction with the native population, since it can expose them to more or less subtle prejudices and discrimination. In this regard, the mentoring relationship did not cause the study participants to reduce their attitude of suspicion and feeling of distrust toward others (*Z* = −1.090; *p* = 0.276). No significant differences were observed by either sex or age.

The perceived availability of social support strengthened, in all cases, the psychological well-being of the mentees. The perception of social support increased and this is reflected in a higher score of the Social Support Index for the whole sample (*Z* = −6.922; *p* < 0.01; *d_Cohen_* = 1.319; Table 2). More specifically, having informal sources of social support correlated negatively with psychological symptoms of stress (Spearman’s rho = −0.337; *p* = 0.000; *d_Cohen_* = 0.716) and positively with the Resilience Index (Spearman’s rho = 0.246; *p* = 0.002; *d_Cohen_* = 0.508), although resilience scores were only significant for the girls (*Z* = −1.661; *p* = 0.097, and as seen when considering a 0.10 error).

After six months, the group of adolescents perceived greater social support from their local network, especially the girls and those under 12 that had more diverse sources of help after the intervention (Table 2). Both of these groups found social support networks in the school and stated having at least one classmate that helped them with the homework (girls group: *Z* = −2.251; *p* = 0.024; *d_Cohen_* = 0.524; under 12 group: *Z* = −2.171; *p* = 0.030; *d_Cohen_* = 0.511; Table 2). This tangible support, which includes physical acts of help and educational support in schoolwork, was not the only kind of support they received. The support of self-esteem, related to recognising their personal worth, was also perceived by these mentees in the school, where they assured: “Someone at school makes me feel successful” (girls group: *Z* = −2.003; *p* = 0.045; *d_Cohen_* = 0.463; under 12 group: *Z* = −1.807; *p* = 0.071; *d_Cohen_* = 0.421; Table 2).

Regarding school dynamics, the results showed that the Nightingale project was associated with more positive attitudes of the mentees towards the school and their peers, specifically for the over-twelves group, who significantly improved their response to the item: “I would prefer to go another school” (*Z* = 2.834; *p* = 0.005; *d_Cohen_* = 0.664; Table 2).

This connection has helped those adolescents who participated in mentoring to improve academically (Table 2). The pre-test–post-test shows an improvement in the Academic Self-Efficacy Index for the participants as a whole (*Z* = −7.693; *p* = 0.000; *d_Cohen_* = 1.548). This improvement is greater in the group of boys (*Z* = −5.905; *p* = 0.000; *d_Cohen_* = 1.778) and in the children under 12 (*Z* = −6.631; *p* = 0.000; *d_Cohen_* = 2.308).

With regard to self-esteem, no changes were observed between pre-test and post-test for the whole sample (*Z* = 0.766; *p* = 0.444; Table 2), nor for boys and girls separately. Statistical significance was only observed in those over twelve years of age, whose self-esteem increased (*Z* = 1.968; *p* = 0.049; *d_Cohen_* = 0.448). However, when controlled for age (under 12 years and over 12 years), mentoring was positively associated with the adolescents’ assessment of their abilities and degree of personal satisfaction. This improvement in self-concept occurred in the oldest participants in the positive responses to the items used in the questionnaires: “I feel that I am a person of worth, at least on an equal level with others” (Z = −2.466; p = 0.014; d_Cohen_ = 0.569) and “I am able to do things as well as most other people” (*Z* = −2.376; *p* = 0.018; *d_Cohen_* = 0.547). This correlates with an improvement in the Cognitive Engagement Index score for the children under 12, the only group in which there are statistically significant differences between the pre-test and post-test scores (*Z* = −2.088; *p* = 0.037; *d_Cohen_* = 0.054; Table 2).

## 5. Discussion

This research aimed to identify whether the presence of a mentor that provides support can improve some specific aspects of the social and emotional well-being of young immigrants. The results of this study show that the development of a mentoring relationship improved some aspects of the psychosocial well-being of young immigrants and refugees, protecting them from the negative impact of the stress involved in adapting to a new country. This finding is consistent with earlier research that shows that the presence of a non-parental adult acts as a support that helps mentees increase their ability to overcome adverse events, such as those arising from leaving and adapting to a new context [26].

The support of a reliable ally provided participants with a number of personal and social benefits insofar as it helped them weave local support networks. Some of these benefits involve an improvement in their emotional and cognitive skills, as well as better social development. The mentees that participated in the project, as has been observed in other participants of formal mentoring programmes aimed at the inclusion of young people of foreign origin [25,59], improved their access to social capital resources, whose social networks constitute the framework in which support exchanges take place. The perceived availability of social support reduced some symptoms of stress associated with the migration process and made the youths more resilient, despite enduring significant adversity. The impact of mentoring on the promotion of resilience in adolescents from ethnic minorities supports the findings of a recent literature review [36,60,61,62].

Participation in the Nightingale project improved the minors’ relationships with their classmates. This corroborates the results of previous research that shows a positive association between mentoring and peer relationship development [63]. The importance of close peer relationships for immigrant adolescents has been found in previous social science research [64,65], the results of which show that peer social support not only promotes adaptation to the host country but also contributes to greater psychological well-being. In the present study, the increase in the perception of social support in school, as well as providing mentees with educational support, strengthened their feeling of personal worth.

The Nightingale project generated significant positive changes in the minors, which helped them in their process of adaptation to the school. During the intervention, the participants improved social relationships with their classmates and also showed more assertive attitudes towards the school and teachers. These results coincide with those found by various authors [13,66], which show the effectiveness of mentoring programmes to promote a positive change in a student’s attitude towards the school. This inner transformation is vital to help adolescent immigrants and refugees to improve their academic performance. Over the course of the programme, the students’ psychological involvement in learning—cognitive commitment—increased significantly. This difference in the pre-test–post-test intervention, as other authors have suggested [3,24], might be due to the positive role model provided by the mentors. The Nightingale project volunteers are young undergraduates who, because of their status as students, can foster the minors’ motivation during their transition to post-compulsory education.

In contrast to other research on informal or ‘natural’ mentoring relationships [46,67], no evidence was found showing the programme’s impact on the reduction in perceived racial discrimination. It is possible that in order for mentors to moderate the attitude of suspicion and feeling of mistrust towards others, the mentor needs to focus on responding to this specific goal—for example, through specific conversations on discriminatory experiences. This was not assessed in the study.

## 6. Limitations

One limitation of this study was that the sample was not randomly selected, which precludes making generalisable statements about mentoring processes that include the immigrant population. This study was based on a single group, pre-test–post-test design, without a control group. The objective was to measure certain changes in the emotional well-being of young people, and it is therefore not an impact analysis of the project as it does not have a comparative reference in a group on which the activity has not been developed. One limitation that should be taken into account is the effect that other non-controlled variables may have had on the observed changes in some aspects of the emotional well-being of young people. The non-experimental pre-test–post-test design does not manage to control confounding variables that might exert some influence on the results. This limitation will be addressed in a second phase of the study, which will incorporate a qualitative analysis of interviews with mentees in which information will be collected about their perception of mentoring in their emotional states. The main difficulty will be that of differentiating the specific effects of the programme from those non-specific effects that derive from the lack of comparability of the group with a control group, which compromises the internal validity of the study. Therefore, causal effects are not deduced from this analysis; rather, the differences in variability in scores need to be understood as possible effects derived from the intervention. In fact, this type of design is not exempt from the Hawthorne effect; that is, the responses may have been induced by the participants’ knowledge that they are being studied.

Nor do the participants represent all the geographical areas where the Nightingale programme is implemented, which means that these results cannot be extrapolated to the mentees that take part in other universities around Europe (Sweden, Norway, Austria, Switzerland, etc.) and Africa (Ghana).

Furthermore, the use of self-report questionnaires structured on Likert-type scales made it difficult to carry out a more precise evaluation, as the participants gave their own meanings and interpretations. While this method allowed a very wide range of effects to be explored—many of which were inaccessible to direct observation—in a relatively short time, the use of this methodology prevented the appearance of emerging categories on the nature of the relationships and their effects. To increase the validity of the measures, we recommend that future evaluations be conducted with multiple sources, for example gathering the testimonies of parents and teachers, and through the use of the multimethod perspective or multiple approaches that facilitate the possibility of studying mentoring relationships quantitatively and qualitatively.

One additional limitation, which could explain the moderate effect of the programme, is the length of the study. Some research points out that the minimum time it takes to build a trusting relationship is six months [68]. It has also been shown that the longer and more trusting the mentoring relationship, the greater the impact capacity of the programmes [69].

Finally, it should be noted that for certain instruments used, the measurement value of their consistency was below the standard values, a fact that that must be taken into account when drawing conclusions, and that necessitates the continued improvement of the instruments applied to the case of mentoring.

## 7. Conclusions

The results of this study provide empirical evidence of the positive effects of social support on the emotional well-being of young immigrants and refugees. Specifically, the potential of mentoring programmes to cushion the stressful events to which they are subjected is made evident. The social support perceived during the Nightingale project contributed to improve some aspects related to the psychological well-being of the mentees, who saw their levels of personal satisfaction increase in a short period of time.

The data gleaned from this study suggest that mentoring is associated with more positive indices of personal well-being, although it can produce different effects in women and men, as a review of the relevant literature has shown [63,70]. While scientific research has increasingly focused on observing the effects of mentoring on social inclusion, empirical work addressing the influence of sex on the process and impact of such a relationship is scarce and mostly comes from studies on school-based mentoring programmes [66,71,72]. Therefore, interdisciplinary work with a gender perspective is needed that can address the gaps that remain in the evaluation of youth mentoring and its effects differentiated by sex.

The results reveal the importance of school counsellors, psychologists and social workers providing immigrant minors with programmes that encourage the building of social networks and the promotion of social support. We believe that prioritising policies and services that ensure a socially supportive environment in the reception of young people of foreign origin may help reduce the stressors associated with the migration process, which place minors in a vulnerable situation. The post-mentoring outcomes support the scientific consensus in the field of youth mentoring regarding the key role that relationships with non-parental adults play in supporting the social inclusion and subjective well-being of young immigrants.

With the aim of promoting the implementation of social mentoring programmes in schools, the findings of this study will be presented to primary and secondary school teachers. Educational institutions must show concern about the reality of students of foreign origin, at risk of exclusion, so as to help them overcome the adversities that arise from the adaptation process, avoiding leaving scars that affect emotional stability and lead to poor academic performance. From what is stated in this study, it can be seen that there is a need to promote the implementation of mentoring programmes as a reception plan that fosters the development of supportive relationships between adults and minors that serve the latter as a resource to try to adapt to the new environment, learn the language, build local networks and plan for the future.

## Figures and Tables

**Table 1:** Composition of the study group.

Study Group	N	Percent
Male	79	50.0
Female	79	50.0
Below 12	77	48.7
12 and above	81	51.3

Source: the authors.

**Table 2:** Non-parametric tests for pre-post measurements.

Measure	N	Mean (Pre)	Mean (Post)	T of Wilcoxon (*Z*)	*p*	*d_Cohen_*
Social Support Index	158	13.66	18.58	−6.922	0.000	1.319
Boys	79	13.37	17.96	−4.534	0.000	1.186
Girls	79	13.96	19.21	−5.243	0.000	1.461
Below 12	77	14.40	19.50	−4.832	0.000	1.319
12 and above	81	12.96	17.72	−4.907	0.000	1.301
Youth Environment Stressors Index	158	2.37	1.53	5.931	0.000	1.07
Boys	79	2.14	1.51	3.666	0.000	0.906
Girls	79	2.61	1.55	4.679	0.000	1.238
Below 12	77	2.14	1.34	4.334	0.000	1.136
12 and above	81	2.59	1.71	4.150	0.000	1.039
Self-Esteem Index	158	30.67	30.37	0.766	0.444	
I have at least one friend at school to help me with homework	158	3.27	3.63	−2.038	0.042	0.329
Boys	79	3.28	3.41	−0.549	0.583	
Girls	79	3.25	3.85	−2.251	0.024	0.524
Below 12	77	3.26	3.81	−2.171	0.030	0.511
12 and above	81	3.27	3.46	−0.713	0.476	
Someone at school makes me feel successful	158	4.01	4.12	−0.706	0.480	
Boys	79	3.89	3.76	0.917	0.359	
Girls	79	4.13	4.48	−2.003	0.045	0.463
Below 12	77	4.09	4.44	−1.807	0.071	0.421
12 and above	81	3.93	3.81	0.698	0.485	
I would prefer to go to another school						
12 and above	81	4.64	4.35	2.834	0.005	0.664
Academic Self-Efficacy Index	158	20.28	22.62	−7.693	0.000	1.548
Boys	79	19.97	22.51	−5.905	0.000	1.778
Girls	79	20.59	22.73	−5.088	0.000	1.396
Below 12	77	20.30	23.27	−6.631	0.000	2.308
12 and above	81	20.27	22.00	−4.178	0.000	1.048
Cognitive Engagement Index	158	9.22	9.48	−1.956	0.051	
Boys	79	9.18	9.33	−0.963	0.336	
Girls	79	9.27	9.63	−1.733	0.083	
Below 12	77	9.21	9.57	−2.088	0.037	0.054
12 and above	81	9.23	9.40	−0.755	0.450	

Source: the authors.

## Data Availability

The data presented in this study are openly available in Harvard Dataserve repository at https://doi.org/10.7910/DVN/ZXC8RC.
